# Effects of Consuming Preloads with Different Energy Density and Taste Quality on Energy Intake and Postprandial Blood Glucose

**DOI:** 10.3390/nu10020161

**Published:** 2018-01-31

**Authors:** Siew Ling Tey, Nurhazwani Salleh, Christiani Jeyakumar Henry, Ciaran G. Forde

**Affiliations:** 1 Clinical Nutrition Research Centre (CNRC), Singapore Institute for Clinical Sciences (SICS), Agency for Science, Technology and Research (A*STAR), National University Health System, Singapore 117599, Singapore; siewling.tey@otago.ac.nz (S.L.T.); n.salleh@massey.ac.nz (N.S.); jeya_henry@sics.a-star.edu.sg (C.J.H.); 2 Department of Biochemistry, Yong Loo Lin School of Medicine, National University of Singapore, Singapore 117596, Singapore; 3 Department of Physiology, Yong Loo Lin School of Medicine, National University of Singapore, Singapore 117593, Singapore

**Keywords:** energy density, non-nutritive sweeteners, umami, glycaemic response, energy intake

## Abstract

Consumption of reduced energy dense foods and drink has the potential to reduce energy intake and postprandial blood glucose concentrations. In addition, the taste quality of a meal (e.g., sweet or savoury) may play a role in satiation and food intake. The objective of this randomised crossover study was to examine whether energy density and taste quality has an impact on energy intake and postprandial blood glucose response. Using a preload design, participants were asked to consume a sweet (“Cheng Teng”) or a savoury (broth) preload soup in high energy density (HED; around 0.50 kcal/g; 250 kcal) or low energy density (LED; around 0.12 kcal/g; 50 kcal) in mid-morning and an ad libitum lunch was provided an hour after the preload. Participants recorded their food intake for the rest of the day after they left the study site. Energy compensation and postprandial blood glucose response were measured in 32 healthy lean males (mean age = 28.9 years, mean BMI = 22.1 kg/m^2^). There was a significant difference in ad libitum lunch intake between treatments (*p* = 0.012), with higher intake in sweet LED and savoury LED compared to sweet HED and savoury HED. Energy intake at subsequent meals and total daily energy intake did not differ between the four treatments (both *p* ≥ 0.214). Consumption of HED preloads resulted in a larger spike in postprandial blood glucose response compared with LED preloads, irrespective of taste quality (*p* < 0.001). Energy density rather than taste quality plays an important role in energy compensation and postprandial blood glucose response. This suggests that regular consumption of low energy-dense foods has the potential to reduce overall energy intake and to improve glycemic control.

## 1. Introduction

Long-term consumption of excess energy in the absence of energy compensation may result in positive energy balance over time, which could lead to higher risk of obesity, cardiovascular disease, diabetes, and certain type of cancers [1,2,3,4]. Numerous aspects of the meal are known to influence the onset of satiation and satiety, and these influences often manifest through the sensory properties of the food being consumed. For instance, food form [5,6,7,8,9], palatability [10,11,12], weight or volume [13,14], energy density [15,16,17,18,19,20,21,22], and portion size [16,23,24,25,26] have been consistently shown to influence energy intake. However, less is known regarding the role of taste qualities in energy intake. 

The sense of taste has a unique role in guiding our food choice. Previous research has estimated that over 85% of the of the energy consumed in the diet is derived from either sweet or salty tasting foods, which contribute to 47% and 39% of energy consumed respectively [27]. Through the use of non-nutritive sweeteners and low calorie savoury tastants (i.e., monosodium glutamate-, inosine-5′-monophosphate, MSG-IMP), today it is possible to accurately match the perceptual “sweetness” of added sugars and the savouriness associated with proteinaceous foods, without incurring the full metabolic impact associated with their consumption. In this regard, it is possible to use reformulation to produce foods and beverages of equivalent sensory appeal, while also moderating the extent to which the consumption of sweet and savoury foods produce deleterious effects on energy balance and health.

The influence of sweet tasting stimuli such as nutritive and non-nutritive sweeteners on energy intake and glycemic control has been studied extensively. Much less is known about the impact of savoury taste stimuli on energy compensation and glycemic control, though both taste qualities tend to have equivalent energy density (4 kcal/g). Currently there is a strong focus on the dangers of passive overconsumption of energy from sweet or high sugar food and beverages, particularly in the form of sugar-sweetened beverages [28,29,30,31,32,33,34,35,36], with the suggestion that refined carbohydrates leave consumers “always hungry” by stimulating insulin release, directing fat towards storage rather than oxidation and stimulating an orexigenic impact on appetite and subsequent food intake [37]. To date, much less attention has been given to directly comparing the satiating properties of energy equivalent sweet and savoury stimuli, and the impact their consumption has on subsequent energy intake.

The current limited evidence suggests that sweet lunches have a less suppressive effect on appetite than non-sweet lunches [38] and savoury taste has a stronger modulating effect on food preference or intake than sweet taste [39,40]. In a double-blind study, MSG was added to a chicken broth and it increased subjective ratings for satiety, promoting feelings of fullness and reducing rated hunger, but the addition did not alter energy intake at the next meal when compared to the same broth without added MSG [41]. Indeed, the impact of MSG on ad libitum energy intake has been mixed, with some studies showing lower energy intake with MSG preloads [42,43] while others reported no effect or higher energy intake with MSG consumption [38,39,40,44,45,46]. Taken together, these results suggest that energy consumed as savoury taste may help to better preserve normal appetite regulation compared to sweet taste, although the impact on later energy intake remains unclear.

Similarly, it is unclear whether taste quality can moderate satiety through postprandial metabolic responses to the energy consumed. Numerous studies have reported the effect of sugar-sweetened beverage [47,48] and non-nutritive sweeteners [49,50] on glycemic control and diabetes risk. By contrast, much less is known regarding the effect of umami taste on blood glucose response. A previous study showed that savoury taste and protein modulated the production of hormones response for food intake regulation, reducing blood glucose and ghrelin while increasing c-peptide and insulin [39]. To our knowledge, no study to date has directly compared sweet and savoury tastes, without the addition of other macronutrients, for their influence on postprandial blood glucose.

Our previous research has shown that energy compensation in response to calorie reduction or supplementation of a covertly manipulated meal is imprecise and energy density of a meal has the potential to promote either positive or negative energy balance [22]. Recently we have shown moderate energy compensation in sweet beverages when the energy from sugar was replaced with non-nutritive sweeteners and sweet taste intensity was maintained [51]. In this study, no statistically significant differences were found in total area under the curve for blood insulin, mean 24-h glucose and 24-h glycemic variability between the test beverages [51,52]. 

The current study aimed to test whether taste quality can influence the degree of energy compensation in similarly realistic food stimuli. The objective of the current study was to determine the effects of energy density (low or high) and taste quality (sweet or savoury) on energy intake and postprandial blood glucose response. We hypothesized that the energy consumed as a “sweet” food would result in a weaker satiety effect than energy consumed as a “savoury” food. Hence energy compensation may be weaker and total energy intake may be higher when energy is consumed as sweet compared to savoury. In addition, we hypothesized that postprandial blood glucose response would be higher for the sweet food compared to an energy equivalent savoury food. Our secondary hypothesis was that energy compensation for higher energy dense preloads would be incomplete and that total energy intake and postprandial blood glucose would be higher for these foods, compared to low energy dense versions of the same food (sweet and savoury).

## 2. Materials and Methods

### 2.1. Study Design and Participants

This study was conducted using a randomised, crossover, 2 × 2 design: sweet vs. savoury, low energy density (LED) vs. high energy density (HED). Participants were randomly allocated to receive the four treatments, with the order balanced, on four test days using an online randomizer (https://www.randomizer.org/). They were required to have a minimum of five-day break between the test days.

A total of 32 male participants were recruited from the general public in Singapore. The inclusion criteria were healthy lean males with a normal BMI (18.5 and 25.0 kg/m^2^) aged between 21 and 50 years. The exclusion criteria were people with intolerances or allergies to test foods, major chronic disease and taking medications known to influence appetite, glucose or energy metabolism. Individuals, who regularly skipped breakfast, were currently dieting or those whose body weight had changed more than 5 kg in the last 12 months were also excluded.

The study was approved by the National Healthcare Group Domain Specific Review Board in Singapore reference number 2015-00749. All study participants provided written informed consent. The trial was registered at Australian New Zealand Clinical Trials Registry, registration code ACTRN12616001128482.

### 2.2. Test Food

The four study treatments comprised of sweet low energy density (LED) (0.11 kcal/g; 9.6 g carbohydrate; 1.1 g protein; 0.7 g fat), sweet high energy density (HED) (0.49 kcal/g; 59.1 g carbohydrate; 1.3 g protein; 0.8 g fat), savoury low energy density (LED) (0.13 kcal/g; 9.6 g carbohydrate; 1.1 g protein; 0.7 g fat), and savoury high energy density (HED) (0.50 kcal/g; 59.1 g carbohydrate; 1.3 g protein; 0.8 g fat) preloads. All four test preloads contained yam glutinous rice balls, sweet potato glutinous rice balls, snow fungus, and corn kernels. The sweet preload soap was developed in the style of a “Cheng Teng” (sweet local dessert) and was sweetened with 0.05 g sucralose. The savoury preload soup was developed in the style of a “cloud mushroom clear savoury broth” and contained 3.6 g of MSG and 1.5 g of IMP. The only difference between the LED and HED preload soups was the addition of 55 g of maltodextrin in the HED preload, and this was the same for both sweet and savoury soups. Both sweet and savoury soups are commonly consumed in Asian countries and all test preloads were spiked with brown colouring in order to mask any potential difference in visual cues between the preloads.

The breakfast and lunch used in the current study are typical foods consumed among Singaporeans and have been used in previous studies conducted on a similar population group at the Clinical Nutrition Research Center (CNRC) [22,51]. The standardised breakfast comprised of a small carton of Milo, an apple, a packet of cheese sandwich biscuits, and a muesli bar (energy content: 529 kcal). The ad libitum test lunch consisted of a glass of water (optional) and 800 g of fried rice (energy content: 1256 kcal).

A pilot test consisting of 12 panelists was conducted to compare the sensory attributes of the preloads before the start of the study. Results showed that the preloads did not differ in thickness (*p* = 0.512), overall flavour intensity (*p* = 0.449), and pleasantness (*p* = 0.260). However, there was a significant difference in sweetness between the treatments (*p* < 0.001), with higher sweetness ratings for sweet LED and sweet HED compared to savoury LED and savoury HED. The opposite was observed for saltiness and savouriness (both *p* < 0.001), in which both savoury (LED and HED) had higher saltiness and savouriness ratings than sweet LED and sweet HED.

### 2.3. Procedure

All participants were asked to attend one screening session and four test sessions. During the screening session, anthropometric measurements, body composition, fasting blood glucose, and blood pressure were collected. Participants were also asked to complete a questionnaire to capture basic health information, food allergies or intolerance, as well as the Dutch Eating Behavior Questionnaire [53].

Participants attended four test sessions, with at least five days between the sessions. Participants were asked to refrain from vigorous physical activity, consume an evening meal with similar macronutrient composition and fast overnight for 10 h. On each test day, participants were asked to consume a pre-package standardised breakfast between 8 a.m. and 9 a.m. They then arrived at the CNRC between 11 a.m. and 12 noon, where they were randomly allocated to receive a fixed portion of one of the four study preloads (451 g for sweet LED, 506 g for sweet HED, 454 g for savoury LED, and 509 g for savoury HED) at baseline (0 min). Participants were asked to consume the entire preload within 15 min. An hour after the commencement of preload consumption (60 min), participants were given an ad libitum test lunch (i.e., fried rice) where they were asked to eat as little or as much as they wished until they were comfortably full within 20 min. Participants consumed the preload and ad libitum lunch in an individual booth. 

Baseline blood samples were taken at 0 min (before preload consumption). Finger prick blood samples were collected directly into HemoCue cuvettes and analysed using HemoCue Glucose 201 analyser for blood glucose analysis. Further blood samples were taken at 15, 30, 45, 60 (before lunch), 90, 120, 150, and 180 min. Participants were allowed to leave CNRC after 180 min and were asked to keep a diet record to note down any foods and drinks they consumed for the rest of the day after they left CNRC (free-living setting). A nutritionist provided verbal instructions to each participant on the way to collect diet records and entered all dietary data. Diet records were analysed using FoodWorks 8 (Xyris Software, Brisbane, Australia).

All participants were asked to complete a diet record and a physical activity questionnaire the day before the test session and on the day of the test session. This information was used to check participant’s compliance to the study protocol. The timings of the breakfast and lunch in this study were chosen based on the diet records reported by a similar population group, i.e., lean healthy young males, in a number of previous trials conducted in our center [22,51,54,55,56].

### 2.4. Hedonic, Sensory and Appetite Ratings

Participants were asked to rate their appetite (i.e., desire to eat something sweet, desire to eat something savoury, hunger, prospective consumption, fullness, and thirst) and mood (i.e., alertness, clear-headed, and happiness) on a 100 mm visual analogue scale anchored with “not at all” (0 mm) and “extremely” (100 mm) at 0 (before preload), 15, 30, 45, 60 (before lunch), 90, 120, 150, and 180 min. In addition to these nine time points, participants were asked to rate the hedonic and sensory attributes of the preload and lunch at two extra time points, i.e., after consuming a spoonful of the preload and a spoonful of the lunch. These attributes include pleasantness, thickness, desire to eat something sweet, desire to eat something savoury, bitterness, sweetness, fullness, familiarity, salty, savoury, and overall flavour intensity. Leftover fried rice and water were weighed and recorded after the meal.

### 2.5. Data Analysis

In order to have 90% power to detect a difference of 30% in total AUC for glucose between the treatments, 25 participants with full data would be required at the end of the study. Total blood glucose response was expressed as the incremental area under the curve (iAUC) above baseline and calculated using the trapezoidal rule. Percentage energy compensation was calculated by subtracting the ad libitum lunch intake for HED preload from ad libitum lunch intake for LED preload and this value was then divided by the difference between HED and LED preloads (i.e., 198 kcal) and multiplied by 100%.

Baseline characteristics of the study participants were reported as means and SD. Linear mixed models were used to compare the effects of the study preload on “appetite”, “energy intake”, “blood glucose response”. Log transformations were performed where this improved residual normality and/or homoscedasticity. The linear mixed models were also used to assess the influence of age, session number, BMI, body fat percentage, resting metabolic rate, fat free mass, lunch intake, and restraint status on all study outcomes. Additional variables such as hunger, pleasantness, and fullness were included in the mixed models for energy intake analyses. None of the variables significantly influenced the study outcomes and hence they were excluded from the final models. Pairwise comparisons between groups were performed when the overall test for differences between groups was significant. No further adjustments were made for multiple comparisons. Marginally statistically significant results should be interpreted with caution. Stata 11.2 (StatCorp LP, College Station, TX, USA) was used for all analyses and two-sided statistical significance was set at *p* < 0.05. 

## 3. Results

### 3.1. Participants’ Characteristics

A total of 34 healthy males were screened for the study and two participants withdrew from the study before randomisation due to personal reasons unrelated to the study. Thirty-two participants completed the study. Participants were relatively young and lean, with a mean (SD) age of 28.9 (7.5) years and a mean (SD) BMI of 22.1 (1.8) kg/m^2^ and mean (SD) body fat percentage of 17.5 (6.3)% (Table 1). Participants’ fasting blood glucose and blood pressure were within the recommendation.

### 3.2. Hedonic, Sensory and Appetite Ratings of the Test Preloads and Lunch

Participants were asked to make hedonic, sensory and appetite ratings after they consumed a spoonful of the test preloads (Table 2). There were small but statistically significant differences in pleasantness and familiarity ratings between the treatments, with the sweet preloads having higher ratings than savoury preloads (both *p* ≤ 0.008). As expected, sweet preloads had significantly higher sweetness ratings compared to savoury preloads whereas the savoury preloads had significantly salty ratings than sweet preloads (both *p* < 0.001).

Participants rated the same attributes after consuming a spoonful of the fried rice for lunch (Table 2). Participants reported feeling significantly hungrier and less full on the days after they consumed sweet LED and savoury LED preloads compared to sweet HED and savoury HED preloads (both *p* ≤ 0.026).

**Figure 1:**
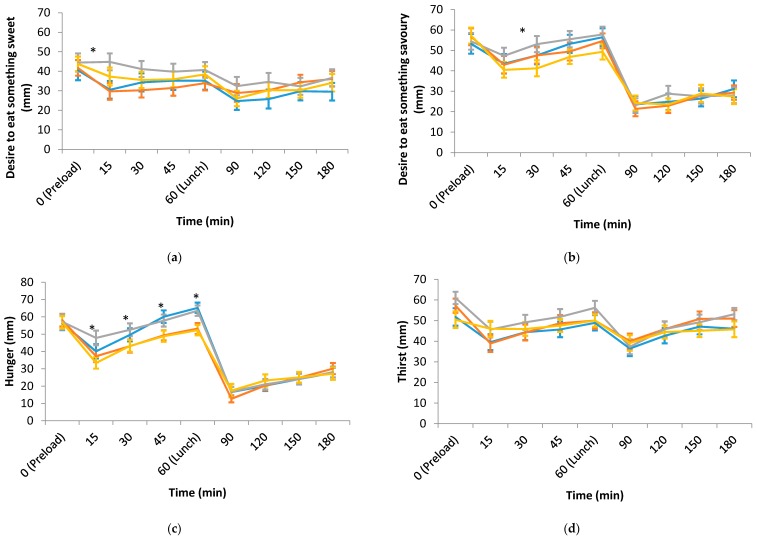

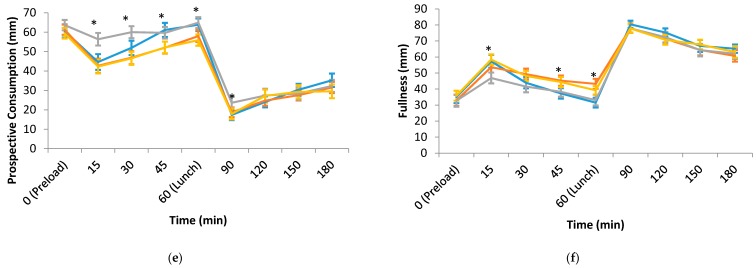
(**a**) Desire to eat something sweet; (**b**) Desire to eat something savoury; (**c**) Hunger; (**d**) Thirst; (**e**) Prospective consumption; (**f**) Fullness ratings over time on each test day (means ± SE; *n* = 32). * Linear mixed models showed statistically significant differences in these appetite measures between the treatments at those time points, *p* < 0.05. Abbreviation: HED, high energy density; LED, low energy density.

Figure 1a–f shows the appetite ratings over a three-hour period on each test day. Hunger and prospective consumption ratings from 15 to 60 min were significantly higher while fullness rating was significantly lower for both LED preloads compared to HED preloads. There were very few differences in desire to eat something sweet, desire to eat something savoury, and thirst between the four treatments across all time points. Similarly, there was very little difference in any of the appetite measures after lunch.

### 3.3. Energy Intake

**Figure 2:**
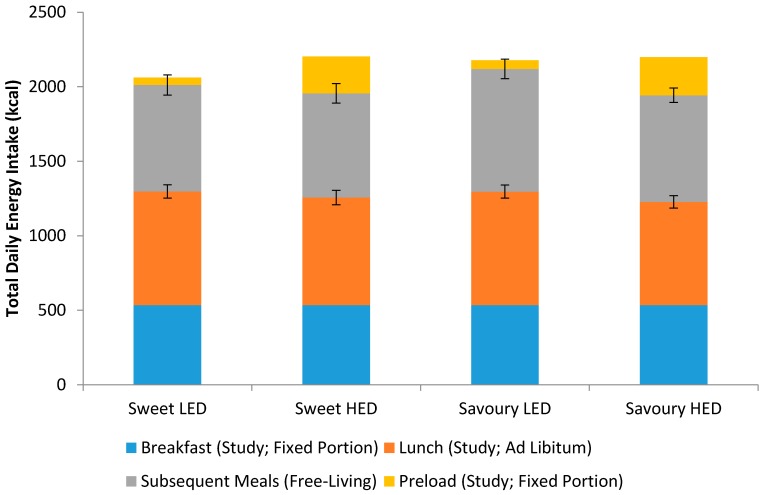
Energy intake consumed at each meal on sweet LED, sweet HED, savoury LED, and savoury HED test days (means ± SE; *n* = 32).

Figure 2 shows the energy intake consumed at each meal on sweet LED, sweet HED, savoury LED, and savoury HED test days. There was a significant difference in ad libitum lunch intake between treatments (*p* = 0.012) with higher intake for both sweet LED and savoury LED compared to sweet HED and savoury HED (Table 2). Ad libitum lunch intake was 763 kcal for sweet LED and 722 kcal for sweet HED, which only accounts for 20.7% of the missing 198 kcal. Ad libitum lunch intake was 762 kcal for savoury LED while 693 kcal for savoury HED and the energy compensation score for savoury LED was 34.8%. Energy intake at subsequent meals (after participants left the study site) and total energy intake were not significantly different between the treatments (both *p* ≥ 0.214).

### 3.4. Postprandial Blood Glucose Response

**Figure 3:**
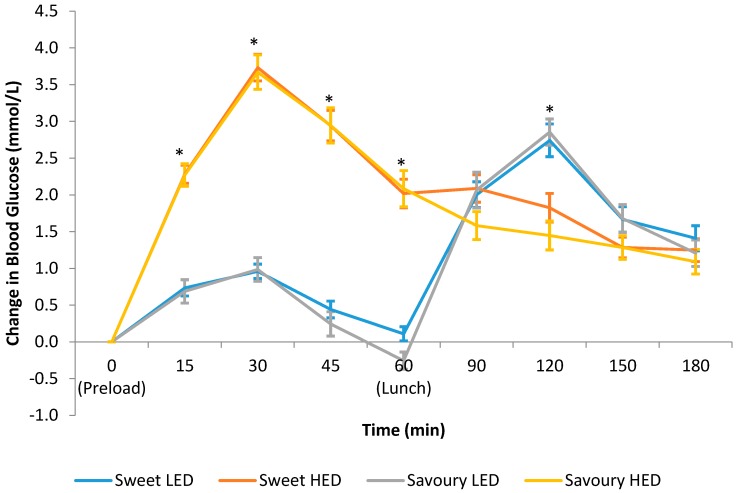
Temporal curves of the blood glucose response for the test preloads (means ± SE; *n* = 30). * Linear mixed models showed statistically significant differences in blood glucose between the test preloads at those time points, *p* < 0.05.

Figure 3 shows the temporal curve of the blood glucose response to the test preloads over a three-hour period. There was a significant difference in blood glucose response between the treatments from 15 to 60 min, with larger spikes in blood glucose over this period after consuming sweet HED and savoury HED compared to sweet LED and savoury LED. On the other hand, blood glucose response was blunted in the first 60 min with both LED preloads but there was a higher rise in blood glucose at 120 min. The iAUC over a three-hour period was 249 mmol/L·min for sweet LED, 355 mmol/L·min for sweet HED, 253 mmol/L·min for savoury LED, and 328 mmol/L·min for savoury HED, which represents a significant difference between the treatments (*p* < 0.001, Table 2). Importantly, there was no significant difference in blood glucose as a function of the taste of the preloads and differences were only seen between HED and LED across both taste conditions.

## 4. Discussion

The present study compared the effects of energy density and taste quality on appetite, energy intake, and blood glucose response. Our results showed minimal differences across all outcome measurements between the sweet and savoury tastes. However, consuming high energy dense preloads suppressed hunger and promoted fullness, which led to lower ad libitum lunch intake compared to low energy dense preloads. Postprandial blood glucose response was significantly higher for the high energy dense preloads, with minimal differences as a function of the taste quality of the energy consumed.

The current study showed that sweet and savoury preloads did not have a specific effect on appetite measures and energy intake. The suggestion that energy consumed in the form of a sweet tasting food is less satiating and promotes greater postprandial hunger and subsequent food intake than savoury foods was not supported by the current findings. Our finding is consistent with previous research reporting no significant differences in appetite ratings or energy intake between sweet and savoury ad libitum meals [40,44]. Although taste quality had no impact on the appetite ratings, hunger and prospective consumption ratings from 15 to 60 min were significantly higher and fullness was significantly lower for both LED preloads compared to HED preloads. Two comprehensive reviews highlighted the importance of the time interval between the preload and test meal [5,57], energy compensation was found to be more accurate when the inter-meal interval was between 30 and 120 min [5]. The time interval between the preload and test meal in the present study was within the recommended range in the previous review [5], i.e., 45 min, and indeed partial energy compensation was observed on both sweet and savoury LED days. 

Several recent reviews reported that substituting the consumption of caloric sweeteners for lower energy dense version of the same foods using non-nutritive sweeteners has the potential to reduce total energy intake and aids in body weight management [33,34,58,59,60,61]. The present study found no statistically significant differences in ad libitum lunch intake, subsequent meal intake (after participants left the study site), and total daily energy intake between sweet LED and sweet HED preloads. The ad libitum lunch intake was 763 kcal for sweet LED and 722 kcal for sweet HED and this only accounts for an average of 41 kcal of the missing 198 kcal within the lunchtime meal, meaning that participants only partially compensated (20.7%) for the “missing calories” when a non-nutritive sweetener was used to replace maltodextrin in the sweet LED preload. These differences tracked for the remainder of the day as total daily energy intake was 2061 kcal for sweet LED while 2203 kcal for sweet HED. This finding highlights an acute imprecise control of energy intake, and provides some support for the current guidelines to substitute nutritive sweeteners with non-nutritive sweeteners in order to reduce overall energy intake [32,62]. Our finding refutes the “conscious overcompensation” hypothesis, which suggests that non-nutritive sweeteners promote overcompensation in later energy intakes, as total daily energy intake remained lower for both lower calorie preload conditions in our study [34,63].

Similarly, the present study showed partial energy compensation at lunch time with savoury LED preload, albeit the magnitude for savoury LED preload was larger than the sweet LED preload, i.e., 34.8% vs. 20.7%. This suggests a slightly more accurate compensation when energy was consumed in savoury form. Although differences in energy compensation appear notable, the difference in overall daily energy intake between sweet HED and savoury HED was negligible (2203 kcal vs. 2199 kcal = Δ4 kcal), indicating that participant’s compensation is primarily in response to differences in preload energy density, and not preload taste quality. Difference in overall daily energy intake was also very small between the two savoury preloads (LED = 2178 kcal vs. HED = 2199 kcal). It is worth noting that the very small differences in overall energy intake across all four conditions are within the margin of error for subjective behavioural measures, and were not significantly different (*p* = 0.214). The present study found small but statistically significant differences in pleasantness ratings between the treatments. When pleasantness of the preload was adjusted for in the mixed models for energy intake analyses, it did not significantly influence the study outcomes (all *p* ≥ 0.543). It appears that pleasantness of the study preloads has minimal influence on energy intake. In addition, energy compensation was relatively consistent for the HED/LED in both sweet and savoury. In this regard, the clearest effect on total energy intake was driven by the partial compensation to preload energy density, rather than their taste quality. 

Preload and ad libitum lunch intake results from the present study are in agreement with previous research, which showed a positive relationship between energy density and overall energy intake [15,16,17,18,19,20,21,22]. Energy intake of preload and ad libitum lunch was 812 kcal for sweet LED, 969 kcal for sweet HED, 820 kcal for savoury LED, and 949 kcal for savoury HED (*p* < 0.001). Using a similar approach, we have recently shown that removing energy from a sweetened drink by substituting sugar with a non-nutritive sweetener produced relatively small changes in daily energy intake, and with no significant net savings in daily energy intake despite the lower calorie preloads [51]. The current study found a similar result where there was no significant difference in total daily energy intake between the four treatments, questioning the impact of small, acute reductions in energy intake on later energy intake, and the need to either increase preload energy deficits or frequency of preload consumption, or extend the intervention period for longer to observe stronger effects on energy intake over time. Similarly, the current study recruited only males in an effort to minimize the variability in energy intake and place the emphasis on the experimental manipulation. Future studies should include both sexes and extend the sampling period beyond a single test meal. Previous research has suggested the effect of MSG on satiety is dependent on the total dose, and it remains unclear whether we could have increased the impact of MSG on satiety by increasing the dosage or number of presentations MSG enhanced samples in the present study [41].

Previous research has shown a modest effect of MSG or savoury taste on appetite ratings suggesting that MSG promoted fullness and reduced desire to eat [38,39]. The current study did not find any significant difference in fullness or hunger feeling following consumption of the savoury preloads compared to sweet preloads. Instead, differences in appetite sensation were driven by the differences in preload energy density, with similar trend for both sweet and savoury at the higher or lower energy density. Similar to previous findings, our findings show the degree of appetite suppression is directly related to the energy density [38]. The present study also examined whether the savoury taste quality of two of the preloads reduced liking and motivation to consume the savoury lunchtime meal through sensory-specific satiety (SSS). Previous studies have shown SSS could occur within two minutes of consumption while a long-term study has shown that SSS effect could last for 12 weeks [64,65,66,67]. Our current study showed no difference in desire to consume the savoury lunchtime meal following sweet or savoury preloads, suggesting that SSS is unlikely to play a role in guiding subsequent energy intake.

The blood glucose response data indicated that blood glucose response was primarily driven by the energy density and carbohydrate amount and was not influenced by the taste quality (savoury vs. sweet). Both HED preloads containing 55 g of maltodextrin resulted in significantly higher postprandial blood glucose response compared to the LED preloads. There has been some suggestion in the past that MSG may lower blood glucose response [39], although in this study the investigators also manipulated protein content, which is known to stimulate incretin release and modulate blood glucose [68]. In the current study, preload energy density was manipulated using the same macronutrient (maltodextrin), specifically to examine whether differences in taste quality could impact postprandial blood glucose response possibly through the cephalic phase response. Our finding confirms that postprandial changes in blood glucose reported previously are likely due to differences in protein content, and less likely the result of the presence of MSG [39], and taste quality has minimal influence on blood glucose response.

Although participants consumed more at lunch and blood glucose concentration was higher 60 min after lunch on both LED test days, the total area under the curve and incremental area under the curve for blood glucose response were statistically significantly higher on both HED test days. These results are consistent with previous studies, which reported that the glycaemic response to maltodextrin was similar to glucose [69,70] and significantly higher than non-nutritive sweetener [71], with the maltodextrin dose ranged from 25 g to 75 g [70,71]. Recent reviews reported that non-nutritive sweetener consumption has minimal impact on postprandial blood glucose response due to the lower carbohydrate load [50] and could potentially be used as a means to improve glycaemic control [49]. In addition, supplementing the diet with MSG in place of NaCl may help to support efforts to reduce salt intake while maintaining food palatability [72]. These results have significant clinical implications, particularly for Asians due to the high prevalence of diabetes in Asia, where Asians tend to develop type 2 diabetes at younger age and lower BMI than Caucasians [73,74,75,76].

## 5. Conclusions

Participants’ appetite response and energy compensation at lunch were primarily driven by energy differences and were less influenced by taste quality. The subsequent meal intake after participants left the study site and total daily energy intake did not differ between the four test treatments. Postprandial glucose response was dictated by the energy density and carbohydrate content of the preload and not its taste quality. The current study is in line with recent findings showing minimal acute changes in overall daily energy intake when the energy density of a sensory matched preload is reduced [51]. Longer term studies are warranted to determine the potential beneficial effect associated with replacing full calorie versions of foods with sensory matched sweet or savoury energy reduced versions of the same foods on net changes in daily energy intake and potential improvements in body weight and glycaemic control.

## Figures and Tables

**Table 1:** Characteristics of study participants (*n* = 32).

Participant Characteristics	Means (SD)
Age (years)	28.9 (7.5)
Height (cm)	172.3 (6.5)
Weight (kg)	65.6 (7.3)
Body mass index (kg/m^2^)	22.1 (1.8)
Waist circumference (cm)	73.9 (5.3)
Hip circumference (cm)	89.4 (3.6)
Bodpod basal metabolic rate (kcal)	1431 (159)
Bodpod fat (%)	17.5 (6.3)
Bodpod fat free mass (%)	82.5 (6.3)
Fasting blood glucose (mmol/L)	4.5 (0.4)
Systolic blood pressure (mmHg)	119.6 (9.6)
Diastolic blood pressure (mmHg)	70.7 (7.3)
All values are means (SD)	

**Table 2:** Hedonic, sensory, appetite ratings, energy intake and glucose AUC for each treatment (*n* = 32).

	Sweet LED	Sweet HED	Savoury LED	Savoury HED	*p* Value
**Ratings after a spoonful of the preload**					
Pleasantness (mm)	46.3 (3.5) ^a,b^	53.5 (3.1) ^b^	37.5 (3.8) ^a^	43.2 (4.0) ^a^	0.008
Thickness (mm)	24.8 (3.5)	27.7 (4.1)	31.3 (4.1)	30.1 (3.8)	0.266
Desire to eat something sweet (mm)	40.7 (4.8)	46.9 (4.6)	46.9 (4.8)	41.7 (4.5)	0.487
Desire to eat something savoury (mm)	51.4 (4.3)	56.8 (3.6)	50.8 (4.4)	51.2 (4.3)	0.475
Bitterness (mm)	22.6 (3.9)	12.0 (2.4)	20.3 (4.3)	18.2 (4.3)	0.076
Sweetness (mm)	53.2 (4.4) ^a^	63.6 (3.5) ^b^	19.5 (3.9) ^c^	19.2 (3.5) ^c^	<0.001
**Expected Fullness** (mm)	48.1 (4.0)	47.2 (3.4)	48.4 (4.0)	51.8 (3.6)	0.627
Salty (mm)	21.0 (3.4) ^a^	27.0 (3.8) ^a^	62.8 (3.7) ^b^	60.7 (4.0) ^b^	<0.001
Savoury (mm)	38.5 (4.8)	37.2 (4.3)	49.2 (3.6)	47.6 (4.3)	0.060
Flavour intensity (mm)	35.7 (3.4) ^a^	49.0 (3.3) ^b^	48.1 (3.7) ^b^	53.8 (3.7) ^b^	<0.001
**Ratings after a spoonful of the lunch**					
Pleasantness (mm)	58.0 (2.6)	59.8 (2.4)	61.0 (3.4)	56.6 (2.9)	0.493
Sweetness (mm)	26.7 (3.6)	27.0 (3.7)	26.8 (3.8)	24.2 (3.8)	0.734
Saltiness (mm)	43.2 (3.1)	44.2 (3.0)	41.5 (3.6)	43.6 (2.8)	0.838
Familiarity (mm)	73.9 (3.0)	74.6 (3.0)	80.1 (3.1)	74.6 (3.3)	0.134
Hunger (mm)	64.6 (3.3) ^a^	58.3 (2.9) ^b^	65.1 (3.0) ^a^	54.7 (3.2) ^b^	0.001
Desire to eat something sweet (mm)	35.5 (4.6)	35.6 (4.0)	35.7 (4.6)	35.8 (4.2)	1.000
Desire to eat something savoury (mm)	58.8 (4.0)	59.5 (3.5)	61.2 (4.0)	54.8 (3.8)	0.236
Prospective consumption (mm)	62.7 (3.4) ^a^	61.9 (3.0) ^a^	65.5 (3.0) ^a^	53.5 (2.9) ^b^	<0.001
Thirst (mm)	45.7 (3.7) ^a^	53.1 (3.5) ^b^	54.7 (3.3) ^b^	49.3 (3.3) ^a,b^	0.029
Fullness (mm)	31.3 (3.1) ^a^	38.1 (2.8) ^a,b^	32.7 (3.2) ^a^	41.4 (3.1) ^b^	0.026
Savoury (mm)	55.6 (3.2)	53.4 (2.7)	52.8 (3.7)	53.8 (3.0)	0.854
Flavour intensity (mm)	51.1 (3.0)	56.3 (2.3)	54.3 (3.5)	52.3 (2.4)	0.407
**Energy intake**					
Ad libitum lunch (kcal)	762.5 (44.4) ^a^	721.7 (48.4) ^a,b^	761.5 (43.5) ^a^	692.8 (41.5) ^b^	0.012
Subsequent meals (kcal)	714.1 (67.3)	698.6 (65.8)	822.7 (65.6)	714.8 (48.6)	0.284
Total daily intake (kcal)	2061 (85.8)	2203 (80.7)	2178 (94.3)	2199 (61.8)	0.214
**Blood glucose response**					
Glucose iAUC 0 to 180 min	249.1 (18.6) ^a^	354.8 (22.3) ^b^	252.5 (18.5) ^a^	327.6 (26.4) ^b^	<0.001
Glucose total AUC 0 to 180 min	1040 (12.9) ^a^	1131 (18.9) ^b^	1043 (13.2) ^a^	1131 (15.2) ^b^	<0.001

All values are means (SE). Rows with different lower-case letters are significantly different from each other (*p* < 0.05). Abbreviation: AUC, area under the curve; HED, high energy density; LED, low energy density.
